# Psychological Stress Exerts Effects on Pathogenesis of Hepatitis B via Type-1/Type-2 Cytokines Shift toward Type-2 Cytokine Response

**DOI:** 10.1371/journal.pone.0105530

**Published:** 2014-08-21

**Authors:** YingLi He, Heng Gao, XiaoMei Li, YingRen Zhao

**Affiliations:** 1 Department of Infectious Diseases, the First Affiliated Teaching Hospital, School of Medicine, Xi'an JiaoTong University, Xi'an, Shaanxi Province, China; 2 Xi'an Municipal Health College, Xi'an, Shaanxi Province, China; 3 Department of Psychology and Nursing, School of Medicine, Xi'an JiaoTong University, Xi'an, Shaanxi Province, China; 4 Institution of Hepatology, the First Affiliated Hospital of Xi'an JiaoTong University, Xi'an, Shaanxi Province, China; Carleton University, Canada

## Abstract

**Background:**

Psychological and physical stress has been demonstrated to have an impact on health through modulation of immune function. Despite high prevalence of stress among patients with hepatitis B virus (HBV) infection, little is known about whether and how stress exerts an effect on the course of hepatitis B.

**Methods:**

Eighty patients with chronic hepatitis B(CHB) completed the Perceived Stress Scale-10(PSS-10) and State-Trait Anxiety Inventory(STAI). Fresh whole blood was subject to flow cytometry for lymphocytes count. Plasma samples frozen at −80°C were thawed for cytokines, alanine aminotransferase (ALT), and virus load. These patients were grouped into high or low perceived stress, state anxiety and trait anxiety groups according to the scale score. Sociodemographic, disease-specific characteristics, lymphocytes count and cytokines were compared.

**Results:**

Firstly, a negative association between ALT and stress (t = −4.308; *p* = .000), state anxiety (t = −3.085; *p* = .003) and trait anxiety (t = −4.925; *p* = .000) were found. As ALT is a surrogate marker of hepatocytes injury, and liver injury is a consequence of immune responses. Next, we tested the relationship between stress/anxiety and lymphocytes. No statistical significance were found with respect to counts of total T cells, CD4+ T cell, CD8+ T cell, NK cell, and B cell count between high and low stress group. Type-2 cytokine interleukin-10 (IL-10) level was significantly higher in high stress group relative to lower counterpart (t = 6.538; *p* = 0.000), and type-1 cytokine interferon-gamma (IFN-γ) level shown a decreased tendency in high stress group (t = −1.702; *p* = 0.093). Finally, INF-γ:IL-10 ratio displayed significant decrease in high perceived stress(t = −4.606; *p* = 0.000), state anxiety(t = −5.126; *p* = 0.000) and trait anxiety(t = −4.670; *p* = 0.000) groups relative to low counterparts.

**Conclusion:**

Our data show stress is not related to the lymphocyte cells count in CHB patients, however, stress induces a shift in the type-1/type-2 cytokine balance towards a type-2 response, which implicated a role of psychological stress in the course of HBV related immune-pathogenesis.

## Introduction

An estimated 2 billion people worldwide are infected with the hepatitis B virus (HBV), and 350 million people are chronically infection[1]. HBV infection results in approximately 280 thousand deaths per year, caused by chronic hepatitis, cirrhosis, and hepatocellular carcinoma, which are associated with heavy psychological stress, enormous medical-care costs,and emotional and economic burden on those afflicted and/or their families[2]. HBV infection is a major life stressor, with up to 90% of those subjects reporting significant stress since the diagnosis of HBV infection[3]. Accumulating evidence has linked stress to the initiation, course and outcome of liver diseases[4]. Emotional stress, such as that induced by hypnotic of fear and anxiety, significantly decreased the hepatic blood flow. Moreover, the type I personality has been associated with the severity of chronic hepatitis C[5]. Stress has been reported to aggravate alpha-galactosylceramide induced hepatitis and carbon tetrachloride induced liver injury[6]. Recently, stress has been implicated in the hepatitis B antibody production in healthy participants[7], however little is known about the role of stress in the course of hepatitis B.

It has been reported that the stress is associated with a suppression of NK cell cytotoxicity[8], lymphocyte proliferation, and the production of IL-2 and IFN-γ, suggesting immunosuppression as a fundamental effect of stress. Moreover, recent data have suggested that dysregulation of type-1/type-2 cytokine balance may play a significant role in stress-associated immune alteration[9]. Type-1 and type-2 cytokines often have opposing actions thought to be critical in maintaining immunologic homeostasis. In the pathogenesis of hepatitis B, it is the host immune response targeting hepatocytes, but not HBV DNA itself, account for liver inflammation and injury. A considerable amount of data suggests that a switch from a predominantly type-1 cytokine-response pattern (e.g., relatively higher levels of IFN-γ) to a predominantly type-2 cell pattern (e.g., relatively higher IL-10) could impact on chronic hepatitis B progression[10]–[12]. Along these lines, we hypothesized that the stress involved in the immune alteration in the course of chronic hepatitis B. To test this, we investigated the association between psychological stress and hepatic inflammation and peripheral lymphocyte counts and circulating type-1 and type-2 cytokines in a series of 80 chronically ill hepatitis B patients.

## Methods

### Subjects

The study was approved by the Research Ethics Committee of the First Affiliated Hospital, Xian Jiaotong University, Shaanxi, China. All subjects signed a written informed consent. This study was performed with the participation of the Departments of Psychiatry, psychology, and Clinical Infectious Diseases between April 2006 and January 2010.

Patients with chronic hepatitis B with positivity for hepatitis B virus surface antigen (HBsAg) more than 6 months were recruited. Diagnoses of chronic hepatitis B were based on clinical, biochemical and virological findings. Criteria for recruitment were: 1) 20∼50 years of age; 2) no cirrhosis by laboratory examination and ultrosonography; 3) AFP<20 ng/ml; 4) no psychosis; 5) no hypercortisolism; 6) no immunological disease and autoallergic disease or a history of illness associated with marked changes in the immune system; 7) no co-infection with other hepatitis virus and no alcoholic hepatitis; 8) without receiving interferon and other immunomodulators within the past one year. A series of 80 eligible patients were included in the study.

### Assessment

All subjects signed a written informed consent. Besides the Stress and anxiety inventory, the subjects were asked to complete a questionnaire that included questions regarding age, sex, education level, Economic status, duration of disease. They subsequently provided disease history, underwent a physical examination, and had a small amount of blood drawn.

Peripheral blood T cell subset measurements were measured with flow cytometry, cell count were determined with 100 µl peripheral blood, using a three-color direct immunofluorescence method. In tube 1, CD3+CD4+ and CD3+CD8+ T cell analyzed with CD4/CD4/CD8 triple staining. In tube 2, B cell and natural killer cell was analyzed with CD16/CD19/CD3 triple staining on a CyFlow SL machine (PARTEC Company, Germany) using FloMax software, as described previously[13].

Plasma samples frozen at −80°C were thawed for cytokine, alanine aminotransferase, ALT, HBV DNA load and genotypes detection as described previously[14]. IL-10 and IFN gamma levels were assayed by using a Quantikine High Sensitivity Immunoassay kit (R & D Systems) according to kit instructions, and all samples from an individual were run in the same way.

### Perceived stress

To obtain information about subjects' stress, we used the 10-item Perceived Stress scale (PSS): an instrument was designed to measure the degree to which situations in one's life are appraised as stressful. This scale assessed the amount of stress in one's life rather than in response to a specific stressor and has been used widely. This scale has good internal consistency (α = 0.80–0.85) and test-retest reliability (r = 0.73–0.85) in Chinese people based on our data. Subjects rated each item from 0 (never) to 4(very often).

### Anxiety

The State-Trait Anxiety Inventory (STAI) provided information on the degree of anxiety. The scales of both State anxiety and Trait anxiety consist of 20 self-report items, with each item running from 1 to 4, for a full score of 80 and a lower score reflecting a better psychological status.

### Statistical analyses

In the present study, we dichotomized chronic hepatitis B subjects into high versus low perceived stress and anxiety score (n = 40/group) on the basis of a median split of the distribution of stress and anxiety score. Based on the scores obtained from these patients, subjects with perceived stress score ≥17 were assigned to the high stress group(P_H_), while subjects with perceived stress score <17 were assigned to the low perceived stress(P_L_); state anxiety ≥41 to the high state anxiety group(S_H_), and <41 to the low state anxiety group(S_L_); trait anxiety score ≥42 to the high trait anxiety(T_H_), and <42 to the low trait anxiety(T_L_).

Chi-square tests, Student-t test, as well as correlation analysis were used for comparing the difference between high and low perceived stress group, high and low state anxiety group and high and low trait anxiety group. SPSS 13.0 statistical software was used for statistical analysis. Reported *p* values were two-sided, and *p* values <0.05 were considered statistically significant.

## Results

### Perceived stress

The Perceived Stress Scale (PSS) is one of most widely used instruments to measure a global level of perceived stress in clinical and research settings[15]. It was reported that 10-item PSS be superior to 14-item PSS[16]. Since this is the first study designed to evaluate the stresses by using PSS-10 for patients with hepatitis B in Chinese population, the reliability of PSS-10 was assessed. The Cronnbach's alpha was 0.88 for the whole scale, the two-week test-retest reliability of PSS-10 was 0.72, suggesting PSS-10 is suitable for evaluating stress in Chinese hepatitis B patients.(During the preparation of this manuscript, the translated Chinese version of PSS-10, has been tested in Chinese policewomen with good internal consistency (α = 0.86) and test-retest reliability (r = 0.68) [17].

Psychological stress lead to emotional reaction of anxiety when an individual perceive stress, therefore the extent of anxiety is associated with the stress level. The State-Trait Anxiety Inventory (STAI) provided information on the degree of anxiety. The scales of Table 1. Sociodemographic, disease-specific characteristics comparison between high and low perceived stress and anxiety patients with chronic hepatitis B.

**Table 1:** Sociodemographic, disease-specific characteristics comparison between high and low perceived stress and anxiety patients with chronic hepatitis B.

Variables^a^	P_H_ (n = 40)	P_L_ (n = 40)	*p* value	S_H_ (n = 40)	S_L_ (n = 40)	*p* value	T_H_ (n = 40)	T_L_ (n = 40)	*p* value
**Gender(F/M)**	8/32	10/30	0.79	8/32	10/30	0.79	10/30	8/32	0.79
**Age, years**	31.15 (8.85)	26.4 (6.01)	0.06	31.20 (8.21)	26.35 (6.85)	0.08	30.80 (8.41)	26.75 (6.88)	0.06
**Education**			0.005			0.050			0.043
<college	32	20		30	22		32	24	
≥college	8	20		10	18		8	16	
**Marital**			0.241			0.241			0.055
Married	28	24		28	24		28	20	
Single	12	16		12	16		12	20	
**Economic status^b^**			0.070			0.459			0.754
≥average	0	4		2	4		2	4	
common	12	16		10	18		14	14	
hard	26	20		28	18		24	22	
**Disease duration^c^**	8.25	7.80	0.080	6.75	9.30	0.06	7.15	8.90	0.100
mean(S.D.)	(6.3)	(5.8)		(5.8)	(6.08)		(5.89)	(6.1)	
**ALT(U/L)**	98.60	138.45	0.000	95.45	132.60	0.003	102.5	137.5	0.000
mean(S.D.)	(44.33)	(55.93)		(47.00)	(53.67)		(43.48)	(56.76)	
**Virus Load^d^**	6.38	6.24	0.18	6.31	6.32	0.28	6.37	6.27	0.26
mean(S.D.)	(1.1)	(1.13)		(1.1)	(1.1)		(1.2)	(1.1)	

a P_H_, high stress group; P_L_, low perceived stress; S_H_, high state anxiety group; S_L_, low state anxiety group;T_H_, trait anxiety score; T_L_, low trait anxiety; ^b^Self reported economic status; ^c^ Years; ^d^ Log_10_(copies/ml).

Both state anxiety and trait anxiety consist of 20 self-report items, with each item running from 1 to 4, for a full score of 80 and a lower score reflecting a better psychological status. Higher scores are positively associated with higher levels of anxiety. To further test the validity of PSS-10, all the participants were encouraged to complete the STAI forms immediately after the completion of PSS-10. Consistency with the conception of psychological stress lead to emotional reaction of anxiety, we do found, a positive correlation between PSS-10 score and state anxiety (r = 0.811, *p*<0.01), and trait anxiety (r = 0.782, *p*<0.01). These results also indicate that psychological stress is associated with mental health issues.

### Sociodemographic comparison between high and low stress/anxiety patients

In China, most of these patients did not fullly covered by medical insurance, and hepatitis B virus infection has been link to substantial economic burden, stigmatization, and poor social support. As far as we know, this is the first study to test the stress of patients with hepatitis B in Chinese population. We want to know if there is association between perceived stress score and sociodemographic variable, clinical variable and virus replication variable. No significant difference was found(Table 1) between high and low stress/anxiety in sociodemographic (gender, age, marital state, and economic state, and disease duration.)

The knowledge of HBV related cirrhosis and hepatocelluar carcinoma and insufficient knowledge of the modes of transmission may results in isolation[3]. Consistently with this conception, in this study, the proportion of high stress scores patients was much higher in inadequate educated participants than well-educated subjects (61.5% *vs* 28.5%), while the proportion of well education was much lower in high stress group than that of the low stress group (20% *vs* 80%, chi-square 7.912, *p* = .009). A positive relationship between education and state anxiety (chi-square 3.516, *p* = .050), and trait anxiety (chi-square 3.810, *p* = .043) were also observed(Table 1).

### Correlation between stress and liver inflammation

**Figure 1:**
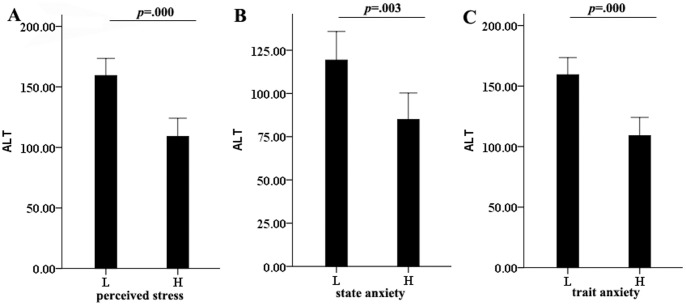
ALT level negatively associated with stress and anxiety. (A)Comparison of ALT between high and low perceived stress group; (B) Comparison of ALT between high and low state anxiety; (C) Comparison of ALT between high and low trait anxiety. *P* value was indicated.

Next, we compared virus load, virus genotypes, and ALT between these two group. As shown in table 1, no differences were observed between high and low stree/anxiety group. However, a strong negative correlation(Figure. 1) between ALT and stress (t = −4.308; *p* = .000), state anxiety (t = −3.085; *p* = .003) and trait anxiety (t = −4.925; *p* = .000) were found. ALT is a surrogate marker of hepatocytes injury. The HBV replication cycle is not directly cytotoxic toward hepatocytes. Much of the liver injury is thought to be a consequence of immune responses. Next, we analyzed the association between the subtypes of lymphocyte and the extent of stress/anxiety. The cell count of T cells (CD3+), T helper/inducer (CD3+CD4+), T suppressor/inducer (CD3+CD8+), NK (CD16+CD56+), B cells (CD19+) in those 80 patients enrolled are comparable with literature[18]. When we compared the cell count between low and high perceived stress group(Table 2), no statistical significance were found with respect to counts of total T cells (t = −0.158; *p* = 0.875), CD4+ T cell (t = 0.648; *p* = 0.519), CD8+T cell (t = −1.810; *p* = 0.074), NK (t = −0.820; *p* = 0.414), and B cells (t = 0.11; *p* = 0.991). Similarly, no statistical significances were found between low and high state anxiety group, low and high trait anxiety in those subtypes of lymphocyte.

**Table 2:** Comparison of lymphocytes count and circulating cytokines between low and high perceived stress patients with hepatitis B.

	CHB with low stress	CHB with high stress	*p* value
CD3	1728 ±812	1605 ± 717	t = −0.158; *p* = 0.875
CD3/CD4	526±243	573 ± 194	t = 0.648; *p* = 0.519
CD3/CD8	519±223	489 ± 213	t = −1.810; *p* = 0.074
CD19	198±98	228 ± 148	t = 0.11; *p* = 0.991
CD16	217±103	197 ± 102	t = −0.820; *p* = 0.414
IFN-γ(pg/mL)	83.48±49.70	62.24±61.35	t = −1.702; *p* = 0.093
IL-10(pg/mL)	9.11±3.36	17.01±6.86	t = 6.538; *p* = 0.000
IFN-γ: IL-10	10.13±4.43	6.23±4.74	t = −4.606; *p* = 0.000

**Figure 2:**
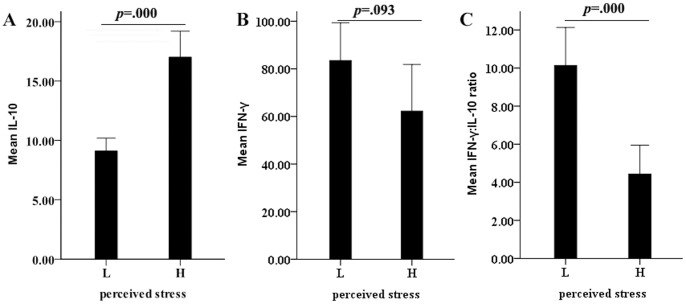
Shift of the type-1/type-2 cytokine balance towards a type-2 cytokine response in high perceived stress patients. (A)Comparison of INF-γ between low and high perceived stress group; (B) Comparison of IL-10 between low and high perceived stress group; (C) Comparison of INF-γ:IL-10 ratio between low and high perceived stress group. *P* value was indicated.

Studies suggest that the balance of cytokine production profiles may play a crucial role in determining the immune response in the liver. Th1 type cytokines (IFN-γ, TNF-*α*) are involved in immune activation and then the injury of HBV antigen expressed hepatocytes, while Th2 type cytokine productions (IL-10, IL-4) inhibit immune reaction and involved in the persistence of infection. In the current study we observed the level of IL-10 was significantly lower in chronic hepatitis B patients with minor stress than patients with major stress(t = 6.538; *p* = 0.000)(Figure. 2A). Since a close correlation between psychological stress and emotional reaction of anxiety was established in our previous data, the correlation between IL-10 and anxiety may be existence. Not surprisingly, IL-10 level was much lower in low state anxiety group (t = 5.821, p = 0.000) and low trait anxiety group (t = 6.238, p = 0.000), as compared with the higher counterpart. We also observed an increased tendency of Th1 cytokine IFN- γ level in high stress group than low stress group, but the statistical difference are not significant(t = 1.702; *p* = 0.093)(Figure. 2B). Similarity with those observations, no significant differences between low and high state anxiety group (t = 1.586, *p* = 0.121), and between low and high trait anxiety group (t = 1.711, *p* = 0.098).

The INF-γ:IL-10 ratio, reflecting the Th1/Th2 balance, has been implicated in psychological stress during an exam period[19]. Next, INF-γ: IL-10 ratio was calculated and then compared between high and low stress/anxiety groups. Consistent with our increased INF-γ and decreased IL-10 data in major stress/anxiety groups as shown above, the ratio displayed significantly increases in high perceived stress(t = 4.606; *p* = 0.000)(Figure. 2C), state anxiety(t = 5.126; *p* = 0.000) and trait anxiety(t = 4.670; *p* = 0.000) groups as compared with the low score groups.

## Discussion

The PSS-10 is one of most widely used instruments to measure a global level of perceived stress in clinical and research settings and has been translated into many languages including Spanish[20], Arabic[21], Greek[22], Japanese[23],Korean[24]. At the beginning of the study, for the reason that this is the first study to employ the translated Chinese version of PSS-10 and STAI inventory scale to assess Chinese population, the reliability and validity were tested. The Cronnbach's alpha was 0.88 for the whole scale, the two-week test-retest reliability of PSS-10 was 0.72, suggesting the translated Chinese version of PSS-10 is suitable for evaluating stress in Chinese hepatitis B patients. The preliminary data were submitted to 13th International Congress on Infectious Diseases as a poster[25]. During the preparation of this manuscript, an independently translated Chinese version of PSS-10, has been tested in Chinese policewomen with comparable consistency and test-retest reliability[17].

Following the validation of the reliability of PSS-10, analysis was conducted in patients with CHB to found the relationship between stress/anxiety and sociodemographic and disease-specific characteristics. Data show stress/anxiety has no association with gender, age, marital state, economic state, disease duration, HBV virus load and HBV gene type. Positively relationship between stress and education was found, Moreover, a negative correlation between stress/anxiety and ALT were established. According to our knowledge, this is the first study discovered the relationship between stress/anxiety and ALT in patients with hepatitis B. The ALT reflects injury to hepatocytes[26]. It was well documented that it's the host immune response targeting infected hepatocytes, not the HBV replication itself in hepatocytes, led to liver injury[27]–[29]. To explorer the role of stress related immune alteration in the course of hepatitis B, we analyzed the stress/anxiety and immune cell count and cytokines, which account for the HBV related immunity response. However, no relationship was found between stress/anxiety and T cells, T helper/inducer, T suppressor/inducer, NK, and B cells count.

Among all the variables assessed, high circulating IL-10 levels was the only variable strongly associated with high stress/anxiety score, low IFN-γ levels was also observed in high stress/anxiety group, however the statistical differences was not significant. IL-10 and IFN-γ are important cytokines involved in chronic HBV infection, as level of IL-10 and IFN-γ associated greatly with course of chronic hepatitis B[30]–[32]. IL-10, a prototype of type-2 cytokine, is considered an anti-inflammatory cytokine and has inhibitory effect on immune response,whereas IFN-γ, a type-1 cytokine, is significantly increased in patients with acute hepatitis B, which would eventually eliminated viral from hepatocytes. Dysregulation of the Th1/Th2 cytokine production is thought to be involved in the pathogenesis of HBV related liver diseases. However, the reasons for the imbalance of Th1/Th2 cytokine still poorly studied in the progression of hepatitis B. Psychological distress has been shown to induce a shift in type-1/type-2 cytokine balance toward a type-2 responses through glucocorticoids and catecholamines. Consistently, we observed a higher IFN-γ/IL-10 ratio, indicating a shift toward a Th2 response, in high stress/anxiety group.

Stress has been reported to aggravate alpha-galactosylceramide induced hepatitis and carbon tetrachloride induced liver injury, however, out data shown stress linked to low ALT level. Thus, a discrepancy between our data and literature was observed. Firstly, stressors are classified into acute, subchronic and chronic. The different duration of stress may elicit different neuroendocrine response and immune alteration. Acute stress may be associated with transient immune activation, while chronic stress seems consistently associated with immune suppression. Most of those studies, including alpha-galactosylceramide induced hepatitis and carbon tetrachloride induce liver injury in animal stress model, the duration of stress was just about weeks. In our study, the diseases duration was around 8 years. Consistent with the suppressive function of chronic stress on immunity, our *in vivo* data shown decreased liver inflammation and injury in high stress/anxiety subjects, as indicated by lower ALT level. Although ALT is a surrogated marker of liver inflammation in the context of hepatitis B and ALT has been widely clinically used, the golden diagnosis of liver inflammation is checking the necrosis of hepatocytes and infiltration of lymphocytes by liver biopsy, which call for further investigation. Secondly, consistent with the immune-suppressive notion of chronic stress, our data presented a markedly increased IL-10, a suppressive cytokine, and a decreased tendency of IFN-γ, an immune activation cytokine. Thirdly, ALT, a marker of liver inflammation, also an indicator of current immune competence, reflects the functional ability of body immunity system mounts a response to HB surface antigen and HB core antigen. It is reasonable that patients with major stress mount less extent of immune response relative to minor stress subjects. Finally, under the basic conception of deleterious effect of chronic stress on health, much attention has paid to the disease severity, while little paid to disease duration. Higher ALT level associated with earlier clearance of virus in both treatment naive patients and interferon treated patients, while lower or normal ALT level associated with persistent infection. In our study, profiles with low ALT, high IL-10, low IFN-γ and low IFN-γ:IL-10 ratio in major stress/anxiety patients is a typical characteristic of persistent infection, while the profiles with high ALT, low IL-10, high IFN-γ and high IFN-γ:IL-10 ratio in minor stress/anxiety patients prone to immunity activation and maybe virus clearance.

To our knowledge, this is the first study to investigate the relation among stress, immunity and hepatitis B. Ours *in vivo* data support the model, at least in part, that alterations in IFN-γ:IL-10 ratio, particular the IL-10 level, secondary to increased psychological stress are involved in the persistent HBV infection.
