# Correction: Psychological Stress Exerts Effects on Pathogenesis of Hepatitis B via Type-1/Type-2 Cytokines Shift toward Type-2 Cytokine Response

**DOI:** 10.1371/journal.pone.0118714

**Published:** 2015-03-05

**Authors:** The PLOS ONE Staff

There is an error in the affiliation of the second author Heng Gao. The affiliation should be: Xi’An Health School, Xi’an, Shaanxi Province, China.
